# Interplay between proteasome inhibitors and NF-κB pathway in leukemia and lymphoma: a comprehensive review on challenges ahead of proteasome inhibitors

**DOI:** 10.1186/s12964-023-01433-5

**Published:** 2024-02-08

**Authors:** Mahdi Pakjoo, Seyed Esmaeil Ahmadi, Mohammad Zahedi, Niloofar Jaafari, Reyhane Khademi, Ali Amini, Majid Safa

**Affiliations:** 1 Department of Hematology, Faculty of Medical Sciences, Tarbiat Modares University, Tehran, Iran; 2 ATMP department, Breast cancer research center, Motamed cancer institute, ACECR, P.O. BOX:15179/64311, Tehran, Iran; 3 Department of Hematology and Blood Banking, Faculty of Allied Medicine, Iran University of Medical Sciences, Tehran, Iran; 4 Department of Medical Biotechnology, School of Allied Medicine, Student Research Committee, Iran University of Medical Sciences, Tehran, Iran; 5 Thalassemia & Hemoglobinopathy Research Center, Health Research Institute, Ahvaz Jundishapur University of Medical Sciences, Ahvaz, Iran

**Keywords:** Proteasome, Proteasome inhibitors, NF-κB, leukemia, Lymphoma

## Abstract

The current scientific literature has extensively explored the potential role of proteasome inhibitors (PIs) in the NF-κB pathway of leukemia and lymphoma. The ubiquitin-proteasome system (UPS) is a critical component in regulating protein degradation in eukaryotic cells. PIs, such as BTZ, are used to target the 26S proteasome in hematologic malignancies, resulting in the prevention of the degradation of tumor suppressor proteins, the activation of intrinsic mitochondrial-dependent cell death, and the inhibition of the NF-κB signaling pathway. NF-κB is a transcription factor that plays a critical role in the regulation of apoptosis, cell proliferation, differentiation, inflammation, angiogenesis, and tumor migration. Despite the successful use of PIs in various hematologic malignancies, there are limitations such as resistant to these inhibitors. Some reports suggest that PIs can induce NF-κB activation, which increases the survival of malignant cells. This article discusses the various aspects of PIs’ effects on the NF-κB pathway and their limitations.

Video Abstract

**Supplementary Information:**

The online version contains supplementary material available at 10.1186/s12964-023-01433-5.

## Introduction

The ubiquitin-proteasome system (UPS) plays a crucial role in the degradation of misfolded, unfolded, or harmful proteins within eukaryotic cells, preventing their accumulation [1]. The UPS consists of two main components: the E1–3 ubiquitin ligases responsible for ubiquitinating substrates and the 26S proteasome, which facilitates protein degradation [2]. PIs such as bortezomib (BTZ) specifically target the 26S proteasome, making the UPS an attractive therapeutic target in hematologic malignancies. The successful use of these drugs in various hematologic malignancies suggests that targeting the UPS could be a promising treatment strategy [3].

Pro-survival proteins and cell proliferation are controlled by the proteasome in both transformed and normal cells. Proteins produced by cancer cells promote cell survival, proliferation, and/or inhibit cell death [1]. On the other hand, inhibitors of the proteasome can prevent tumor-suppressor proteins from degrading [4]. BTZ, carfilzomib, and ixazomib are three PIs that have been approved by the US Food and Drug Administration (FDA) [1]. It has been suggested that PIs may interact with anti-apoptotic proteins like Bcl-xL and Bcl-2 to activate intrinsic mitochondrial-dependent cell death [1, 5, 6]. They also suppress the nuclear factor κB (NF-κB) signaling pathway through preventing the degradation of inhibitory κB (IκB) proteins in the proteasome [7].

PIs impair tumor growth through a variety of mechanisms, including blocking the degradation of IκBα, a negative regulator of NF-κB, through the proteasome. NF-κB is a transcriptional factor with anti-apoptotic properties renowned as a key survival factor in various malignancies [8–10]. It regulates apoptosis, cell proliferation, and differentiation as well as inflammation, angiogenesis, and tumor migration through transcription of growth factors/signaling molecules (IL-6, TGFb, TNFa, IGF-1, SDF-1, HGF), cell-adhesion molecules (VLA-4, VLA-5, ICAM), angiogenesis factors (VEGEFs, angioprotein-1, MCP-1), and anti-apoptotic enzymes (Bcl-2, Bcl-XL, cIAP, XIAP, FLIP, STAT-3, and Mcl-1) [11–14].

The NF-κB family operates in classical, alternative, and atypical pathways [15]. Activated by various factors, NF-κB normally resides in the cytoplasm bound to IκB proteins [16, 17]. Phosphorylation triggers IκB degradation, allowing NF-κB entry into the nucleus. Consequently, PIs can impede IκB degradation, thereby inhibiting canonical NF-κB activity. Furthermore, the conversion of p50 from its precursor protein p100 also relies on proteasome activity; thus, PIs can additionally hinder the non-canonical pathway [18].

The introduction of first- and second-generation proteasome inhibitors has significantly improved the outcomes for patients diagnosed with multiple myeloma (MM) and mantle cell lymphoma (MCL). However, relapses are common, and over time, patients develop acquired resistance to the treatment emerges [1, 19, 20]. Interestingly, there have been reports suggesting that PIs, despite their original intended purpose, may actually induce activation of NF-κB [21]. As mentioned earlier, NF-κB is known to promote the survival of malignant cells. In this article, we delve into the various aspects of how PIs affect the NF-κB pathway in hematologic malignancies.

## Overview on NF-κB pathway

The transcription factor NF-κB, a member of the NF-κB family, plays an essential role in regulating cellular processes such as proliferation, differentiation, and programmed cell death or apoptosis [22]. Dysregulation of the NF-κB [23] pathway has been observed in inflammatory and immune disorders, as well as in hematological malignancies [24]. The aberrant activity of NF-κB has been linked to the chronic inflammation-cancer transformation connection, where it suppresses apoptosis, enhances cellular proliferation, facilitates cell migration and invasion, stimulates angiogenesis, and promotes metastasis, among other effects [25]. Various factors including bacterial and viral infections, necrotic cell products, oxidative stress, DNA damage, and pro-inflammatory cytokines can activate NF-κB. The activation of the NF-κB pathway involves the activation of multiple signaling cascades [26].

RelA (p65), NF-κB1 (p50; p105), NF-κB2 (p52; p100), c-Rel, and RelB1,2 are structurally related members of the mammalian NF-κB family that bind to a specific DNA element to target genes [27]. These proteins possess a conserved N-terminal region called the Rel Homology Domain (RHD), which spans approximately 300 amino acids. The RHD encompasses domains responsible for dimerization, nuclear localization, and DNA binding. Among its multiple functions, the RHD facilitates dimerization and binding to DNA, as well as interaction with IκB proteins and translocation to the nucleus. In addition to the RHD, RelA, RelB, and c-Rel proteins contain a non-homologous transactivation domain at their C-terminus. This domain significantly enhances transcription by facilitating binding to NF-κB sites. However, certain Rel proteins, such as p50 homodimers, function as transcription repressors despite lacking the transactivation domain. These p50 homodimers can bind to κB-consensus sites and inhibit transcription [28]. The p50 and p52 proteins are generated by proteolytic processing of their precursors, p105 and p100, respectively. With the exception of RelB, all members of the NF-κB family have the ability to form homodimers as well as heterodimers. The most common activated form of NF-κB consists of a heterodimer composed of the p65 subunit and either the p50 or p52 subunit. In contrast, RelB expression is predominantly found in specific regions of the lymph nodes, thymus, and Peyer’s patches. Similarly, c-Rel is primarily expressed in hemopoietic and lymphoid cells. The transcription of c-Rel, RelB, and p105 is regulated through the activity of NF-κB.

The IκB protein family consists of seven members, namely IκBα, IκBβ, IκBγ, IκBε, Bcl-3, and the precursor proteins P105 and P100. All IκB proteins share a common characteristic of having ankyrin repeat domains (ARD), which are repetitive sequences of approximately 30 amino acids. These domains serve as interaction sites for proteins and can undergo proteolytic cleavage and degradation [29]. When an IκB protein binds to an NF-κB dimer, it directly interacts with specific protein sequences in the Rel Homology Domain (RHD) through these ankyrin repeats. This binding inhibits the activity of the NF-κB dimer through two main mechanisms. Firstly, it disrupts the function of the nuclear localization sequence (NLS), resulting in the retention of NF-κB in the cytoplasm. Secondly, it impedes NF-κB’s ability to bind to DNA, thereby blocking its transcriptional activity. Bcl-3, unlike other IκB proteins, exhibits an atypical role by selectively binding to p50 and p52 homodimers, promoting the expression of genes regulated by κB sites [29]. In some instances, such as with IκBζ and Bcl-3, the NF-κB-IκB complex can still bind to DNA, with the IκB protein acting as a transcriptional co-activator. NF-κB protein dimers are essential for the migration of transcription factors to the nucleus, where they interact with DNA. In the inactive state, the IκB protein acts as a specific inhibitor of NF-κB by binding to the RHD, thereby preventing NF-κB activation [29]. Ankyrin repeats in the inhibitory proteins IκBα, IκBβ, IκBγ, IκBε, Bcl-3, precursor proteins P105 and P100 bind to the RHD; therefore, cells must first remove NF-κB proteins from their inhibitors in order to activate NF-κB molecularly [30]. As the NF-κB dimer enters the nucleus, two main signaling pathways are activated, resulting in the dissociation of the inhibitory protein IκB from the NF-κB dimer. Activation of the IκB kinases (IKK) in these signaling pathways, results in the phosphorylation of inhibitory proteins IκB, which is followed by ubiquitination and degradation by the proteasome [31–33]. Ubiquitination of IκB leads to the translocation of NF-κB from the cytoplasm to the nucleus, where it stimulates the transcription of particular cellular genes [34]. It has been suggested that the activation of the NF-κB pathway is involved in the pathogenesis of chronic inflammatory diseases such as rheumatoid arthritis, asthma, and inflammatory bowel disease [35]. Furthermore, alteration in NF-κB regulation may also be associated with other diseases such as Alzheimer’s disease and atherosclerosis, which are both characterized by an inflammatory response to some extent [29, 36]. Additional abnormalities in the NF-κB pathway are frequently observed in a variety of human hematologic cancers, including leukemia and leukemia [37, 38]. Various signaling pathways can activate NF-κB, including those triggered by cytokines, growth factors, and tyrosine kinases. Overexpression of receptors such as tumor necrosis factor receptor (TNFR), insulin growth factor receptor (IGFR), and epidermal growth factor receptor (EGFR) can contribute to the activation of NF-κB. Moreover, the activation of NF-κB can also be mediated by other signaling pathways, such as the phosphatidylinositol 3-kinase and serine/threonine protein kinase B (PI3K/Akt pathways) and the Ras/mitogen-activated protein kinase (Ras/MAPK pathways). These pathways play a role in relaying signals that ultimately lead to the activation of NF-κB [24, 37, 39].

**Fig. 1:**
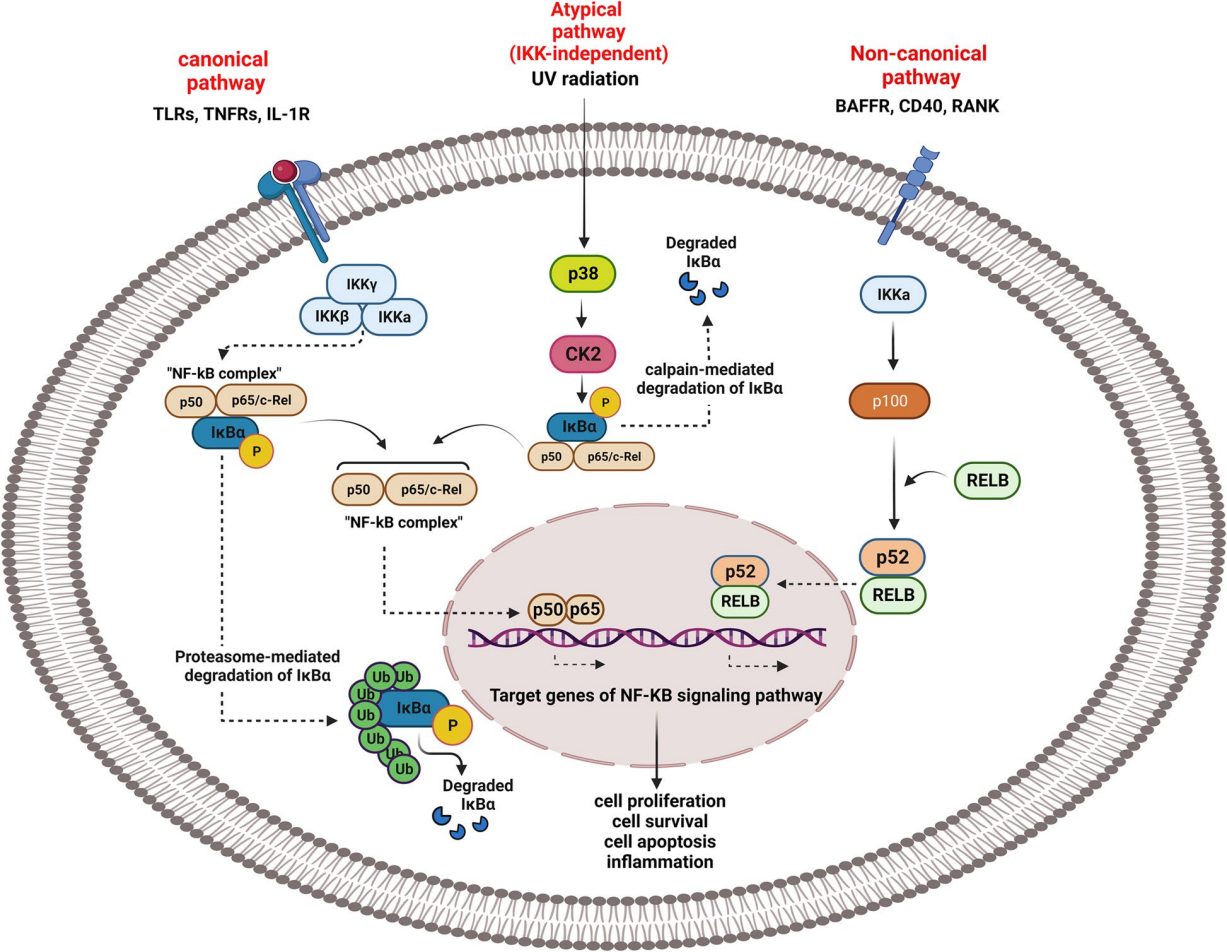
The canonical and non-canonical pathways of the NF-κB signaling pathway. This signaling pathway comprises three distinct routes: the canonical, non-canonical pathways and atypical pathway. In the canonical pathway, Toll-like receptors (TLRs), tumor necrosis factor receptors (TNFRs), and interleukin-1 receptors (IL-1Rs) are activated, leading to the phosphorylation and subsequent degradation of the inhibitory protein IκB. As a result, NF-κB is liberated from the complex with IκB and translocates to the nucleus. Conversely, the non-canonical pathway relies on the activation of the NF-κB2 (p100)/RelB complex by specific receptors such as B-cell activating factor receptor (BAFFR), CD40, and receptor activator of nuclear factor κB (RANK). This activation triggers a cascade of events, including the phosphorylation of NF-κB-inducing kinase (NIK), which in turn phosphorylates IKKα. Consequently, the p52-RelB heterodimer is activated and translocates to the nucleus. The activation of NF-κB signaling pathway exerts regulatory effects on various cellular processes by controlling the expression of cytokines, chemokines, and other genes. Several “atypical” pathways have been described, including radiation-induced NF-κB activation. NF-κB activation follows an atypical pathway, involving distinct phosphorylation events such as Tyr42 phosphorylation by Syk or Src family kinases, triggered by various stimuli. This leads to IκBα dissociation from the RelA/p50 dimer without proteasomal degradation. In the PEST domain, CKII phosphorylates IκBα at Ser293, promoting calpain-mediated degradation

**Fig. 2:**
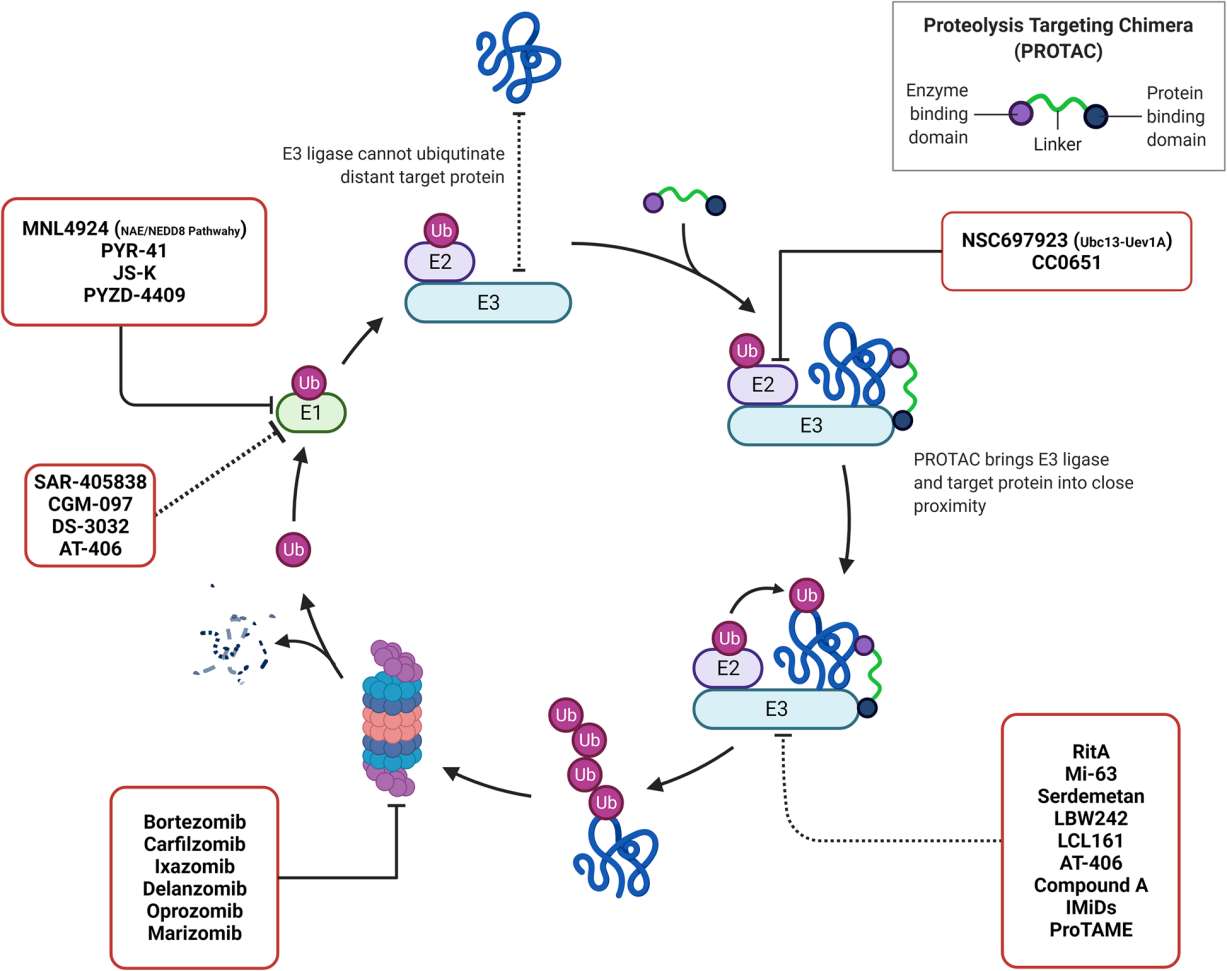
Overview on agents targeting different parts of proteasome-mediated degradation pathway, including E1, E2, E3, and the proteasome itself

NF-κB activation encompasses three primary signaling pathways: the canonical, non-canonical (or alternative), and atypical pathways (Figs. 1 and  2). While these pathways exhibit distinct signaling mechanisms, both the canonical and non-canonical pathways play crucial roles in regulating immune and inflammatory responses. The activation of the canonical pathway is initiated by cell surface receptors, including Toll-like receptors (TLRs), tumor necrosis factor receptors (TNFRs), and T/B cell receptors, in response to pro-inflammatory cytokines and pathogen-associated molecular patterns (PAMPs). These receptors initiate a cascade of signals within the canonical pathway [26, 38]. Upon binding of the ligand molecules to these receptors and subsequent signal transduction across the cell membrane, the IKK complex is activated. The predominant form of this complex is a heterodimer consisting of IKKα (IKK1) and IKKβ (IKK2) catalytic subunits, along with an IKKγ (also known as NF-κB essential modulator or NEMO) regulatory subunit. Once activated, the IKK complex phosphorylates IκB (specifically at Ser32 and Ser36 of IκBα), induces polyubiquitination (specifically at Lys21 and Lys22 of IκBα), and facilitates its subsequent degradation via the 26S proteasome. This process is primarily mediated by IKKβ in an IKKγ-dependent manner [32, 37, 40]. Upon release from the inhibitory IκB proteins, the NF-κB dimers, typically consisting of p50-RelA subunits, undergo translocation into the nucleus. Once inside the nucleus, they bind to specific DNA sequences and initiate the transcription of target genes. This canonical pathway plays a crucial role in activating innate immune responses, promoting inflammation, and inhibiting apoptosis, thereby contributing to the regulation of various biological processes [41]. Another NF-κB activating pathway is the non-canonical or alternative pathway. For example, CD40 (Tumor necrosis factor receptor superfamily member 5), Lymphotoxin β-receptor (LTβR), B-cell activating factor receptor (BAFFR), and Receptor activator of nuclear factor κ B (RANK) are all ligands of a subset of the tumor necrosis factor receptor superfamily (TNFR) members that are activated by the non-canonical NF-κB pathway [27]. This signaling pathway operates independently of the IKKβ and IKKγ dimers, instead relying on the IKKα dimer. Notably, the NF-κB-inducing kinase (NIK) plays a crucial role in this pathway by activating and collaborating with IKKα to facilitate the phosphorylation of p100. This phosphorylation event leads to the ubiquitination and subsequent degradation of p100. It is proposed that NIK phosphorylates and activates the IKKα complex, which in turn phosphorylates p100, enabling the release of active p52/RelB heterodimers. In this pathway, IKKα homodimers primarily target the transcription factor NF-κB2/p100, specifically phosphorylating it at two C-terminal sites [32, 42]. Furthermore, in addition to polyubiquitination and proteasomal degradation, phosphorylation of specific sites is crucial for the conversion of p100 to p52. Unlike the complete degradation of IκB proteins, phosphorylation-dependent ubiquitination of p100 leads to partial degradation of its inhibitory C-terminal region while preserving the N-terminal region. Consequently, the N-terminal portion of NF-κB, which contains the Rel Homology Domain (RHD), is released. As the RHD of p100 is commonly associated with RelB, activation of this “alternative” pathway results in the nuclear translocation of p52–RelB dimers. Subsequently, these dimers bind to DNA, initiating the activation of downstream gene transcription [36, 38].

The atypical pathway of NF-κB activation involves distinct phosphorylation events. Tyrosine (Tyr42) phosphorylation at the N-terminus of the IκBα inhibitor or serine phosphorylation within its PEST domain (rich in proline, glutamic acid/aspartic acid, serine, and threonine residues) triggers this pathway [43–45]. Tyr42 phosphorylation is regulated by spleen tyrosine kinase (Syk) in response to Nerve Growth Factor (NGF) or Ciliary Neurotrophic Factor (CNTF) stimulation, or by members of the Src family of tyrosine kinases in response to Brain-Derived Neurotrophic Factor (BDNF) activation. This phosphorylation leads to the dissociation of IκBα from the RelA/p50 dimer. Unlike in the canonical pathway, liberated IκBα is not degraded by the proteasome [43–45]. In the PEST domain, casein kinase II (CKII) can phosphorylate IκBα at Ser293. Serine phosphorylation promotes the calpain-mediated degradation of IκBα. Additionally, several “atypical” pathways have been described, including radiation-induced NF-κB activation. DNA double-strand breaks, which occur as a result of ionizing radiation, can activate NF-κB signaling through ATM-dependent processes. ATM-mediated activation of IKK via NEMO/IKKγ leads to the phosphorylation and proteolytic degradation of IκBα, followed by nuclear translocation of the RelA(p65)/NF-κB1(p50) heterodimer. Another non-canonical mechanism involves the activation of the DNA sensing adaptor STING by DNA breaks, which depends on IFI16 and ATM. This activation leads to the activation of the innate immune response through NF-κB activation [43–45].

The activation of NF-κB is tightly regulated in normal cells, where it is only activated in response to specific stimuli, leading to the upregulation of its target genes. Following activation, regulatory mechanisms ensure that NF-κB returns to its inactive state, making the activation process reversible and transient. However, certain molecular alterations in tumor cells can disrupt the normal regulation of NF-κB activation. As a result, NF-κB loses its inducibility and becomes constitutively activated [46]. This dysregulated activation leads to abnormal gene expression controlled by NF-κB. These genes are involved in crucial processes such as cell cycle control, apoptosis, migration, and adhesion. Given the importance of these processes in cancer progression and development, there is a clear association between NF-κB and carcinogenesis. The constitutive activation of NF-κB contributes to the dysregulation of gene expression and promotes tumor development and progression [46].

Regarding the regulatory role of NF-κB in apoptosis, intrinsic (mitochondrial) and extrinsic cell death suppression may be facilitated by several NF-κB target genes [28]. Proteins that prevent cell death may be increased in NF-κB-activated cells; therefore, the FLICE-like inhibitory protein (FADD-like IL-1β-converting enzyme-inhibitory protein or FLIP) could be mentioned as an example. Unlike caspase-8, FLIP is not a protease and competes with caspase-8 for binding to the death-inducing signaling complex (DISC) [47]. Consequently, high concentrations of FLIP inhibit the recruitment of caspase-8 to DISC. In addition to being upregulated in a variety of tumors, FLIP has been found to be associated with tumor resistance to death receptor-mediated apoptosis [48]. NF-κB also stimulates the production of inhibitors of apoptosis (IAPs) and some members of the anti-apoptotic Bcl-2 family. The IAPs (c-IAP1, c-IAP2, and XIAP) suppress apoptosis induced via both extrinsic and intrinsic pathways by directly inhibiting effector caspases (caspases-3, − 6, − 7, and 9) and indirectly inhibiting caspase-3 [24, 27, 28].

NF-κB transcription factors contribute to cell survival by influencing the expression of specific pro-survival BCL-2 family members, particularly BCL-XL and A1/BFL-1. These results suggest that blocking NF-kB signaling, such as inhibiting IKK, an upstream activator of NF-kB, could potentially improve the efficacy of BH3-mimetic drugs or chemotherapeutic agents in killing cancer cells. This would occur by reducing the levels of pro-survival BCL-2 proteins. The BH3 domain is a critical part of pro-apoptotic proteins, and it interacts with anti-apoptotic BCL-2 family members to induce cell death. BH3-mimetic drugs are synthetic compounds that mimic the BH3 domain and selectively inhibit the anti-apoptotic BCL-2 proteins [49, 50]. By doing so, they promote apoptosis in cancer cells, which often evade normal cell death mechanisms. These drugs are of interest in cancer therapy because they can counteract the overexpression of anti-apoptotic proteins that contribute to the survival and resistance of cancer cells. By blocking these proteins, BH3-mimetic drugs aim to restore the normal apoptotic process and enhance the effectiveness of other cancer treatments, such as chemotherapy. They are being explored as potential components of combination therapies for various types of cancers. BH3-mimetic drugs may be used in combination with proteasome inhibitors like BTZ to target multiple pathways involved in cancer cell survival and apoptosis regulation. The goal is to improve the overall therapeutic response and overcome potential resistance mechanisms in cancer cells [49, 50].

Another role of NF-κB in cancer progression is to regulate proliferation and invasion. Cyclins D1, D2, D3, and E,, and c-myc are some of the cell cycle-related genes which regulated by NF-κB to promote cell cycle progression [28]. NF-κB also increases the expression level of Intercellular Adhesion Molecule 1 (ICAM-1), Endothelial-Leukocyte Adhesion Molecule 1 (ELAM-1), and matrix metalloproteinases (proteins involved in the invasion). Moreover, NF-κB stimulates the production of a variety of angiogenic factors, such as vascular endothelial growth factor (VEGF), which acts as a critical role in the development of malignancies [25]. Several studies have been conducted to identify potential NF-κB inhibitors as cancer therapeutic agents. Since the activation of NF-κB is the result of a multi-step signaling pathway, these compounds may target different points along the signaling process. One of the approaches to inhibiting NF-κB signaling is to target the proteasome degradation process. Since the NF-κB activation is dependent on the degradation of IκBα, inhibiting the proteasome that degrade IκBα could also be used as pharmaceutical intervention. Therefore, PIs block the degradation of NF-κB1/p105 or NF-κB2/p105, IκBs, and thus prevent NF-κB activation [36, 37, 46].

## Proteasomal degradation pathway

### Structure and functions

The capacity of cells to modify their protein quantities in accordance with fluctuating environmental circumstances is vital for their survival. The processes of protein synthesis, folding, and breakdown all contribute to the maintenance of protein levels. Eukaryotic cells utilize a range of pathways to facilitate the degradation of proteins [51], among these pathways, the lysosomal pathway holds significant importance, while the cytosolic pathway stands as the second major pathway. Within the cytosolic pathway, there exists a specialized structure known as a proteasome.

The proteasome, a substantial protein complex present in eukaryotes, archaea, and certain bacteria, has exhibited remarkable conservation throughout evolution. It has been recognized as a fundamental element of a crucial mechanism through which cells regulate the levels of specific proteins and eliminate misfolded or damaged proteins that pose a threat to cellular integrity. This process necessitates metabolic energy to execute its functions effectively [52, 53]. The proteasome comprises a 20S core particle (CP) and two 19S regulatory cap particles, collectively forming a 26S complex with a molecular mass of around 700 kDa, also known as PA700. The central portion of the proteasome is composed of a cylinder divided into four heteroheptameric rings. Two of these rings are positioned in the center, while the other two encircle the cylinder, forming the outer α-rings and the inner β-rings. The catalytic centers responsible for protein degradation are located within this central cylinder part [54].

Within each of the two β-rings, there are seven β subunits, resulting in a total of 14 β subunits in the proteasome. These β subunits collectively host three active protease sites in each ring. Consequently, a mature eukaryotic proteasome possesses a total of six proteolytic sites, exhibiting three distinct types of proteolytic activities. The cap, responsible for regulating the entry of proteins, is attached to the outer rings of the proteasome [55]. The outer α-rings include seven identical but unique α-subunits that act as a tightly controlled “gate” for the admission of substrates and the elimination of degradation products from the complex by producing a pore [56].

The proteasome’s hollow core forms an enclosed compartment where proteins are targeted for degradation. At each end of the core particle, there is an associated 19S regulatory subunit that contains multiple ATPase active sites and ubiquitin-binding sites. This regulatory subunit plays a crucial role in recognizing polyubiquitinated substrates. It unfolds the targeted proteins, removes the ubiquitin molecules attached to them (deubiquitination), and translocates the unfolded proteins into the catalytic core. Within the catalytic core, the proteins are degraded into smaller fragments called oligopeptides [54, 57].

The ubiquitin protein is composed of 76 amino acids and possesses a highly conserved sequence. It functions by forming a covalent attachment to the target protein, thereby marking it for degradation. The ubiquitin molecule acts as a signal, directing cellular proteins towards the ATP-dependent 26S proteasomes for subsequent degradation [58]. Ubiquitin has seven lysine residues where other ubiquitin molecules can attach, forming various types of polyubiquitin chains. The specific lysine residue involved is crucial. To deliver substrates to the 26S proteasomes for degradation, three types of enzymes (E1, E2, and E3) are involved in tagging substrate proteins with ubiquitin chains. The polyubiquitin chain acts as a signal, guiding target proteins to the proteasome for proteolytic breakdown. The system involves multiple enzymes (two E1 proteins, around 30 E2 proteins, and over 500 species of E3 in humans) to accurately select proteins for degradation [59, 60].

In the first step, a ubiquitin-activating enzyme (E1) hydrolyzes ATP and adenylates a ubiquitin molecule. After activation, E2 (ubiquitin carrier protein or ubiquitin-conjugating enzyme [USC]) transports ubiquitin from El to a substrate attached to a ubiquitin-protein ligase, E3 [60]. Ubiquitin ligases (E3) recognize the specific protein to be ubiquitinated and catalyze the transfer of ubiquitin from E2 to this target protein. By repeating the above steps, other ubiquitin molecules are added to the target protein to form a polyubiquitin chain linked together by lysine 48 [61]. Proteins with at least four ubiquitin monomers in the form of a polyubiquitin chain are recognized by proteasome caps [62]. Following ubiquitination, a protein is identified by the 19S regulatory particle in an ATP-dependent binding phase. Ubiquitin must be eliminated before tagged proteins reach the proteolytic core of proteasomes [62].

Degradation takes place within the central chamber formed by the association of the two rings and does not typically release partially degraded products, instead of reducing the substrate to short polypeptides typically 7–9 residues long. Each catalytic subunit also contains a conserved lysine residue required for proteolysis [63]. For the substrate protein to interact with the proteolytic active sites, it needs to reach the interior of the 20S particle. However, to access the center of the 20S particle, the substrate must undergo partial unfolding. This unfolding of the substrate is necessary for translocation, which refers to the movement of the unfolded substrate into the core of the 20S particle. It’s important to note that translocation occurs after the process of deubiquitination, where the ubiquitin molecules attached to the substrate are removed [64, 65].

The atomic structure of the substrate-engaged 26S proteasome in the deubiquitylation-compatible state suggests that substrates must be unfolded approximately 20 amino acid residues before translocation. However, substantial tertiary structure, particularly nonlocal interactions such as disulfide bonds, is sufficient to inhibit degradation [58]. The gate formed by the α subunits prevents peptides longer than about four residues from entering the interior of the 20S particle [64]. Prior to translocation, the ATP molecules that were bound during the initial recognition step undergo hydrolysis. The energy provided by ATP hydrolysis is required for the unfolding of the substrate but is not necessary for translocation itself. In certain cases, similar to the NF-κB complex in mammals, certain transcription factors are initially synthesized as inactive precursors. These precursors undergo ubiquitination and subsequent degradation by proteasomes, leading to their activation [66]. Such action necessitates the proteasome cleaving the substrate protein internally rather than degrading it from one end. Long loops on the surfaces of these proteins may function as proteasomal substrates and enter the central cavity, while the remainder of the protein stays outside. Similar effects have been seen in yeast proteins; ubiquitin/proteasome-dependent processing (RUP) controls this selective degradation mechanism.

### The interplay between NF-κB and proteasome

**Fig. 3:**
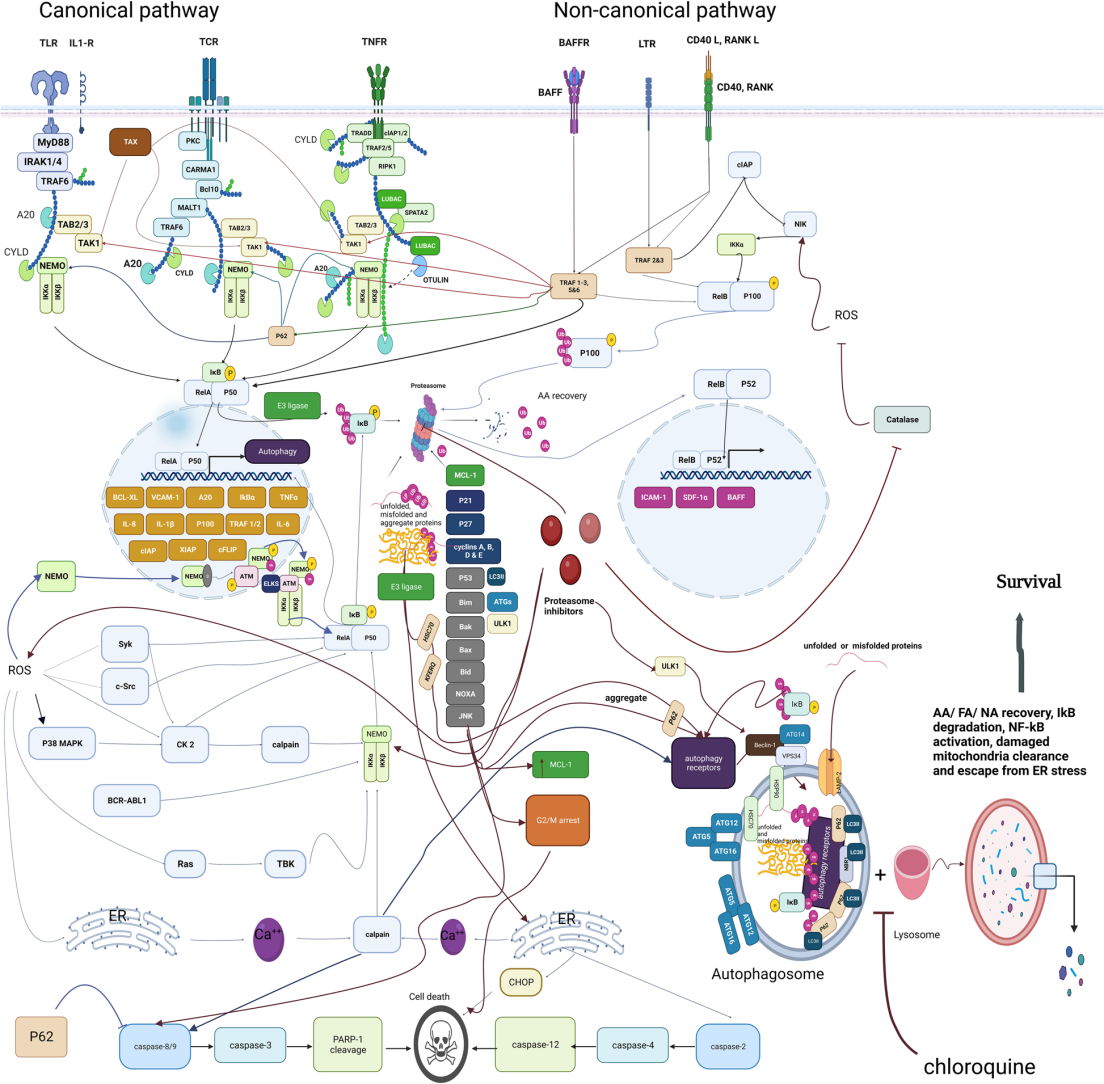
Autophagy’s dual-edge effect on NF-KB and PI resistance. In a physiological context, autophagy can impede the progression of malignancies by eliminating accumulated oncoproteins and inducing autophagic cell death. NF-KB, on the other hand, is primarily activated through canonical and non-canonical pathways, promoting cell survival during cellular stress conditions. PIs have the ability to hinder both of these NF-KB activating pathways, leading to apoptosis induction. However, various mechanisms that stimulate autophagy can give rise to resistance against PIs and cell death by degrading IKK-B and activating NF-KB

Inflammation is an innate defense mechanism that responds to physical, physiological, and oxidative stress. It involves the activation of the canonical NF-KB signaling pathway, which is conserved in all multicellular species. This pathway plays a critical role in coordinating the immune response and regulating inflammation-related gene expression, contributing to the body’s defense against stressors and maintenance of homeostasis [67]. NF-κB is a dimeric pro-inflammatory transcription factor required for normal cell function. Studies have shown that this signaling pathway regulates cell adhesion, differentiation, proliferation, autophagy, cell survival, and apoptosis (Fig. 3) [59].

NF-κB isn’t a single gene but a family of closely linked transcription factors—NF-κB1 (p50/p105), NF-κB2 (p52/p100), RelA (p65), c-Rel, and RelB—producing seven proteins with Rel Homology Domains (RHD) [67, 68]. RHD is crucial for dimerization, inhibitor interaction, and DNA binding. Activation occurs through two pathways: canonical (by pro-inflammatory cytokines like TNFα and IL-1) and noncanonical. While essential for immune responses, NF-κB’s elevated activity in cancers stems from mutations, oncogenes, or increased cytokine release in the tumor microenvironment [69]. It plays a dual role in cancer by both defending against and promoting tumorigenesis. In quiescent cells, NF-κB is sequestered in the cytoplasm by IκB proteins— IκBα, IκBβ, IκBɛ, and Bcl-3—preventing translocation to the nucleus. Phosphorylation of IκB proteins by the IKK complex (IKKα, IKKβ, and IKKγ) activates NF-κB [24, 70].

Upon phosphorylation, the IκB proteins undergo proteasome-dependent degradation. The β-transducin repeat-containing protein (βTrCP) ubiquitinates the phosphorylated IκBs, marking them for degradation by the 26S proteasome. This degradation process leads to the release of NF-κB heterodimers from IκBs, allowing their translocation into the nucleus. In the nucleus, NF-κB binds to κB responsive elements and promotes the transcription of target genes involved in various cellular processes [24, 70]. NF-κB can be activated through multiple signaling pathways that are triggered by various cytokines, growth factors, and tyrosine kinases. The increased expression of receptors such as the epidermal growth factor receptor, insulin-like growth factor receptor, and tumor necrosis factor receptor families has been implicated in the activation of NF-κB. These receptors initiate signaling cascades that lead to the activation of NF-κB, contributing to its enhanced activity [28].

NF-κB has been identified as a critical indicator of cancer, as supported by evidence showing increased expression of numerous NF-κB target genes in various tumors. This upregulation of NF-κB target genes in cancer cells can hinder apoptosis, enhance cell proliferation, and facilitate the invasion of cancer cells, underscoring the potential consequences of NF-κB dysregulation [71]. Due to the multi-step nature of NF-κB activation through signaling pathways, different compounds have the ability to target various stages of this signaling process. For instance, certain anti-inflammatory drugs can impede NF-κB activation by interfering with the activity of IKK, thereby providing a means to regulate NF-κB function [72]. Another way to approach NF-κB inhibition is to target the process of proteasome degradation. PIs prevent NF-κB activation by blocking the degradation of IκBs, NF-κB1/p105, or NF-κB2/p100 [28].

#### Proteasome inhibition and its effect on NF-κB

As mentioned earlier, the inhibition of proteasome activity has been found to exhibit anticancer effects through multiple mechanisms. These include disrupting the progression and control of the cell cycle, inducing apoptosis, and suppressing cell proliferation and angiogenesis [73]. PIs work by destabilizing the 26S proteasome (Fig. 2), a complex responsible for breaking down intracellular proteins. This disruption prevents the degradation of important proteins involved in essential cellular functions. By interfering with these processes, PIs effectively hinder cancer cell growth and survival. Their ability to impede the degradation of key intracellular proteins contributes to the inhibition of cancer cell proliferation and promotes anti-cancer effects [73, 74].

PIs have demonstrated the ability to induce cell death and impact multiple pathways utilized by cancer cells. One potential mechanism underlying their cytotoxic effects involves the inhibition of the NF-κB pathway, which plays a critical role in promoting cell survival, particularly in hematopoietic lineages [75]. When the proteasome is inhibited, IκBα remains unaltered and continues to bind to the p50/p65 NF-κB heterodimer, preventing the activation of the NF-κB pathway. Initially, the inhibition of NF-κB signaling was considered to be the primary mechanism underlying the anti-cancer effects of PIs, as this pathway is involved in critical cellular processes such as cell proliferation, invasion, metastasis, and angiogenesis. However, it has been observed that a potent inhibitor of IκB kinase, known as PS-1145, which blocks NF-κB activation upstream of IκBα degradation, does not exhibit the same cellular toxicity profile as PIs. This suggests that there may be additional mechanisms, besides NF-κB inhibition, that play an equally or potentially more important role in mediating the anti-cancer effects of PIs [76].

Extensive research has primarily focused on the ability of PIs to target the 20S proteolytic core of the proteasome [1, 77]. Three PIs have been approved for the treatment of MM or mantle-cell lymphoma (MCL). The first of these inhibitors, BTZ, was the initial PI to be clinically utilized. Several theories have been proposed to explain how these drugs suppress NF-κB signaling, specifically by inhibiting IκB degradation, which subsequently prevents the translocation of NF-κB. BTZ, classified as a first-generation PI, was developed based on this underlying rationale. Subsequently, second-generation agents such as carfilzomib, ixazomib, and oprozomib were developed, with the aim of improving upon the therapeutic benefits observed with BTZ [1, 77]. Since its FDA approval in 2003 and 2006 for MM and relapsed or refractory MCL (R/R MCL), respectively, BTZ has played a significant role in the treatment of hematologic malignancies, particularly in patients with MM and MCL [78]. This milestone marked the beginning of a new era in the development of PIs for cancer treatment. Subsequently, two more PIs, carfilzomib and ixazomib, have received approval for clinical use [79]. Ongoing research continues to explore the potential of other agents and combination therapies involving PIs in the treatment of hematologic malignancies. These advancements have significantly improved treatment outcomes for patients with these types of cancers.

BTZ, peptide boronic acid and a slowly reversible inhibitor of the β5 catalytic subunit, binds with the catalytic site of the 26S proteasome, enabling inhibition of the β5/chymotrypsin-like and, to a lesser extent, the β2/trypsin-like and β1/post-glutamyl peptide hydrolyzing activities. BTZ demonstrated impressive clinical activity in treating R/R MM in phase I and II studies conducted over the past decade. Generally, it is administered as an intravenous bolus (IV) or subcutaneous injection (SC) in first-line and R/R cases of MM or MCL [80]. As a result of extensive studies conducted on patients with relapsed MM, the FDA accelerated the approval of BTZ in 2003, specifically for the treatment of patients with refractory disease. Subsequently, in 2005, full regulatory approval was granted based on the positive outcomes observed. BTZ has shown significant improvements in various clinical parameters, including time to progression (TTP), overall response rate (ORR), and overall survival (OS), for patients who have been treated with it. These findings have contributed to establishing BTZ as an important therapeutic option for patients with MM [1, 77].

Blocking NF-κB activity is a crucial outcome of BTZ’s mechanism of action, and it likely plays a role in several of the mentioned effects. NF-κB is responsible for stimulating the expression of growth factors, cell adhesion molecules, angiogenesis, and anti-apoptotic factors such as Bcl-2. By inhibiting NF-κB, BTZ disrupts these processes, leading to reduced cell growth, impaired adhesion, inhibited blood vessel formation, and increased susceptibility to apoptosis. These actions contribute significantly to the overall impact of BTZ in treating various conditions [81].

BTZ has been demonstrated in previous studies to significantly inhibit NF-κB DNA-binding activity and nuclear translocation in chemosensitive and chemoresistant MM cell lines. So, in general, proteasomes initiate this pathway by degrading NF-κB inhibitors. Activating this pathway is beneficial for cancer cells and increases growth and survival [81]. In hematologic malignancies, NF-κB is significantly increased. When we use the PI and inhibit the proteasome, the NF-κB pathway inhibitor remains active, the pathway is inhibited, and inflammation is prevented. So, we disable it so that the cell cannot function normally. The proteasome normally inhibits NF-κB inhibitors, such as IKK, to keep the NF-κB pathway active. Several clinical trials have examined the effects of PIs, particularly BTZ, on patients with other hematological malignancies, including acute myeloid leukemia (AML), myelodysplastic syndrome (MDS), and acute lymphoblastic leukemia (ALL). These responses, however, did not warrant further investigation [80, 82] .

To address the limitations of BTZ, several second-generation PIs have been developed. These inhibitors exhibit distinct chemical structures, biochemical properties, binding affinities, reversibility, potency, and selectivity. One class of inhibitors, known as epoxyketones, shares a similar mode of action with boronic acids like BTZ and ixazomib. Epoxyketones can bind to the N-terminal threonines present in the catalytic subunits of the proteasome, which are involved in the cleavage of peptide bonds. Specifically, the α-, β-epoxyketone moiety of these inhibitors forms irreversible morpholino adducts by binding to both the hydroxyl group and the free α-amino group of Thr1 in the catalytic β subunits of the proteasome [83].

Currently, this class includes the most specific and potent PIs. As a PI, carfilzomib (CFZ) is an irreversible peptide epoxyketone class that can prolong proteasome inhibition for extended periods. Despite its high specificity for proteasomes, carfilzomib exhibits minimal activity against other enzymatic and receptor/ligand targets. Compared to BTZ, epoxyketone-based PIs have a longer duration of activity because fresh subunit synthesis and proteasome assembly are needed to restore proteasome activity [1, 83].

In 2012, the FDA approved carfilzomib as a single agent to treat MM in patients who had received at least two prior lines of therapy before and whose disease had remained refractory to the most recent therapy. Like BTZ, carfilzomib causes apoptosis by activating intrinsic and extrinsic caspase pathways [84].

Several studies have shown that carfilzomib exhibits significant cytotoxic activity against various cell lines and patient cells associated with myeloma, lymphoma, leukemia, and solid tumors. Additionally, carfilzomib plus docetaxel reduced tumor growth statistically significantly in a lung carcinoma model compared to the single agents alone. In a colorectal carcinoma model, carfilzomib plus liposomal doxorubicin significantly reduced tumor growth [83, 84].

As a result of carfilzomib administration, endothelial dysfunction has been proposed by inhibiting endothelial nitric oxide synthase activity. Patients who will be receiving carfilzomib are advised to undergo an echocardiography assessment prior to beginning treatment. However, the utility of this assessment in predicting cardiac events has yet to be proven, and further research is necessary in this area. It is also essential to monitor closely for shortness of breath, lower extremity edema, and paroxysmal nocturnal dyspnea. Additionally, carfilzomib has an infrequent tendency to cause renal impairment, even rarer with BTZ. These differences indicate the need for further research into the apparent differences between these two drugs’ downstream effects [84].

Ixazomib, Delanzomib, Oprozomib, and Marizomib are some of the newer second-generation PIs being developed in clinical trials. Similarly, to carfilzomib, oprozomib is an irreversible peptide epoxyketone PI under investigation. Ixazomib is a reversible PI made from the boronic ester. It is the first oral PI entering clinical trials in patients with MM. MM patients with significant pretreatment R/R disease who receive single-agent Ixazomib report clinical activity with infrequent peripheral neuropathy [80, 85].

The second-generation PI Delanzomib (DLZ) is also reversibly binding boronate-based. It is both oral and intravenous bioavailable. In hematologic and solid tumor cell lines and primary cells of MM patients, DLZ showed proteasome-inhibitory activity similar to that of BTZ. With linear PK, the drug’s half-life was rather long, 62 hrs. Myeloma, lymphoma, and solid tumors are currently being studied with DLZ intravenously. In a phase I study of patients with advanced solid tumors and MM, DLZ showed a favorable safety profile and minimal neurotoxicity [80].

The only non-peptide-based inhibitor in clinical trials is marizomib, an irreversible PI. In contrast to other PIs, marizomib inhibits all three catalytic subunits of the 20S proteasome rapidly, broadly, and persistently [86]. Marizomib is administered intravenously twice a week and is being tested in Phase Ib for recurrent MM, solid tumors, lymphoma, and leukemia. There has been a response to marizomib in patients with BTZ-refractory MM. A lack of treatment-emergent PN, myelosuppression, or thrombocytopenia has been reported with marizomib, unlike BTZ [86]. It has now been well established that PIs as a therapeutic intervention in cancer are on the verge of clinical development. Our ability to target the UPP in future years will likely continue to expand, as proteasome inhibition is one of several approaches available to alter UPP function in anti-tumor therapeutic approaches [87] (Table 1).

**Table 1:** PIs utilized in hematologic malignancies

PI	Binding type	Subunit Inhibition	Descriptions (Target residue/ Administration Route/ History of FDA)	Clinical Trials	
BTZ (Velcade) (PS-341) [88]	Reversible [89]	ß5 and LMP7 [88] Chymotrypsin-like activity ß5 and β1-subunit in immunoproteasomes as caspase-like activity at lower affinity [90, 91].	Subcutaneous and Intravenous (IV) [92] First synthesized in 1995 [6] FDA: May 2003 for relapsed/refractory MM [93] 2006 for MCL [94]	NCT01248923	MM, PCL
				NCT00006362	Leukemia, Lymphoma, MM, PCL
				NCT00303797	Refractory CLL, Refractory MM, Stage III MM, Stage IV CLL
				NCT00295932	Leukemia, Lymphoma
				NCT00440765/ NCT00440479	MM, Hematological Neoplasms
				NCT00038571	R/R B cell lymphoma
				NCT00477412/ NCT00633594/ NCT01504776	MCL
				NCT00901147	Recurrent/ Refractory Peripheral T-cell Lymphoma, NK/T-cell Lymphoma
Carfilzomib (Kyprolis) (PR-171) [88]	Irreversible [88]	ß5 and LMP7 [88] both ß5 and ß2 [90] β5, LMP2 and MECL1 subunits of the immunoproteasome [88] Proteasome subunit ß5 [95] ß8, ß1, ß9, ß2, ß10: [96]	Threonine residue [88] Intravenous [90] FDA: July 2012 [97]	NCT01658904/ NCT04065789	MM, PCL
				NCT00150462	WM, NHL, HD, MM
				NCT01470196	WM
				NCT01276717	Relapsed / Recurrent/ Refractory Lymphoma
				NCT01775553/ NCT03029234/ NCT04811508/ NCT00603447	Relapse/ Recurrent/ Refractory MM
				NCT01949532	Relapse MM, End-stage renal disease
				NCT01949545	Solid Tumors, Hematologic Malignancies, Hepatic Impairment
				NCT02145403	Hematologic Malignancies, Relapse GVHD
				NCT02142530	NHL, DLBL, FL, Peripheral T-cell Lymphoma
				NCT00721734	MM, Renal Insufficiency
				NCT01926665	Lymphoma
				NCT01336920	Adult Nasal Type Extranodal NK/T-cell Lymphoma, Anaplastic Large Cell Lymphoma, Angioimmunoblastic T-cell Lymphoma, Peripheral T-cell Lymphoma, Recurrent Adult T-cell Leukemia/Lymphoma
				NCT00884312	MM, Solid Tumors
				NCT01212380	B- CLL, Hematopoietic/Lymphoid Cancer, PLL, Recurrent SLL, Refractory CLL
				NCT01137747	Relapsed AML or ALL
				NCT02095834/ NCT01903811	Recurrent/ Refractory Plasma Cell Myeloma
				NCT02491359	Chronic GVHD
				NCT02187133	Lymphoma,NHL
				NCT01204164	AML, ALL, Blast Crisis of CML,MDS, MM
				NCT02551718	Recurrent Refractory Acute Leukemia of Ambiguous Lineage, Recurrent Refractory ALL, Recurrent Refractory AML
Ixazomib citrate (Ninlaro) (MLN9708) (MLN 2238) [88]	Reversible [89]	ß5 and LMP7 [88] β5 site, at higher concentrations, also seems to inhibit the proteolytic β1 and β2 subunits: [90, 98]	Threonine residue [88] Oral [90, 99] IV, Oral [100] FDA: November 2015 [101]	NCT02169791	Acute Leukemia, Chronic Leukemia, MDS, Lymphomas, MM
				NCT02070458	Recurrent/ Refractory Adult AML
				NCT00893464	Lymphoma
				NCT03323151	Active, not recruiting: Relapsed/Refractory MCL
				NCT04079738	Active, not recruiting: Relapsed/Refractory AML, Adult AML
				NCT03082677	to Prevent Recurrent or Late Acute and Chronic GVHD 1-year After Allogeneic HSCT in Patients With Hematologic Malignancies
				NCT01939899	Relapsed and/or Refractory FL
				NCT01830816	MM, Advanced Solid Tumors
				NCT01953783	Advanced Solid Tumors, Lymphoma
				NCT02057640	MM, Kahler Disease, Plasma-Cell Myeloma, Myelomatosis
				NCT02250300	Allogeneic HSCT
				NCT02513498	Chronic GVHD
				NCT02504359	Relapsed High-Risk MM, PCL, Recurrent PCL
				NCT01912222	Advanced Solid Tumors, Hematologic Malignancies
				NCT02400437	WM
				NCT02158975	Relapsed/Refractory Cutaneous and Peripheral T-cell Lymphomas
Oprozomib (ONX-0912) (PR-047) [88, 97]	Irreversible [88, 90]	ß5 [88, 90] LMP7 [88]	Threonine residue [88] Oral [100] Orphan drug by the U.S. FDA for the treatment of rare type of blood cancer (Waldenstrom’s macroglobulinemia) https://myelomaresearchnews.com) analog of carfilzomib	NCT02072863	MM
				NCT02939183	Active, not recruiting: R/R MM
Marizomib (natural product, named salinosporamide A) (NPI-0052) (MRZ) [97, 100]	Irreversible, Sustained [97, 100]	All three proteolytic subunits (β1, β2 and β5) [90, 100]	Threonine residue [100] IV (Oral and Subcutaneous efficacy in vivo) [100]	NCT00629473/ NCT00461045	MM
				NCT02103335	R/R MM
				NCT00667082	Non-Small Cell Lung Cancer, Pancreatic Cancer, Melanoma, Lymphoma, MM
				NCT00396864	Cancer, Lymphomas
Delanzomib (CEP-18770) (CIP 18770) (CT 47098) (NPH-007098): [97]	Slowly reversible [100] Irreversible: [99]	Chymotrypsin-like and Caspase-like activities of the proteasome (β5 and β1) [85, 90]	Threonine residue [100] IV (Oral efficacy in vivo) [100]	NCT00572637	Solid Tumors, NHL
				NCT01023880	Phase I/II Study for Relapsed Refractory MM [102]
(KZR-616) a derivative of ONX-0914: [88]	Irreversible [88, 103]	LMP2 and LMP7 [88]	Threonine residue [103]	NCT03393013	Lupus Nephritis Systemic Lupus Erythematosus
M3258: [104]	Reversible [89]	Highly specific inhibitor of LMP7 (ß5i) [89, 105]	Threonine residue [105] Oral (animal model) [89]	NCT04075721	MM
ONX0914 (PR-957) [90, 104, 106]	–	Inhibits > 95% of LMP7 (ß5i) and 60–80% of LMP2 (ß1i) activity of the immunoproteasome: [104]	- - - - - -	–	–
LU-102 [104]	–	MECL1 and ß2c [104]	–	–	–

Abbreviations: *MM* Multiple myeloma, *PCL* Plasma cell leukemia, *CLL* Chronic lymphocytic leukemia, *MCL* Mantle cell lymphoma, *WM* Waldenstrom’s macroglobulinemia, *NHL* Non-Hodgkin lymphoma, *HD* Hodgkin disease, *GVHD* Graft vs. Host Disease, *DLBL* Diffuse large B-cell lymphoma, *FL* Follicular lymphoma, *PLL* Prolymphocytic leukaemia, *SLL* small lymphocytic lymphoma, *AML* Acute myeloid leukemia, *ALL* Acute lymphocytic leukemia, *MDS* Myelodysplastic syndromes, *HSCT* haematopoietic stem cell transplantation

### Non-PI inhibitors of the UPS

In addition to the proteasome, UPS components play an essential role in cancer progression, and small molecule inhibitors consistently have sound effects on tumor suppression. Here, we summarize the current clinical investigations on inhibitors of ubiquitinating enzymes.

MLN4924 is a small molecule that inhibits the NEDD8-activating enzyme (NAE) E1 enzyme. It is an adenosine sulfamate analog that binds covalently to NAE, forming a NEDD8-MLN4924 adduct. This adduct blocks the neddylation process in all cullin-RING ligases (CRLs), which regulate the degradation of various intracellular proteins. MLN4924 has been tested in phase I clinical trials for specific solid tumors and hematologic malignancies, and it has demonstrated clinical effectiveness in a phase I trial involving acute myelogenous leukemia [107, 108].

SAR-405838, a compound jointly developed by Ascenta Therapeutics and Sanofi, CGM097, developed by Novartis, and DS3032b, developed by Daichi Sankyo, are all designed to target the interaction between MDM2 and p53. These compounds are undergoing phase I clinical trials for patients with advanced solid tumors, either as standalone treatments or combined with chemotherapy [107].

A small compound known as NSC697923 inhibits the movement of Ub (ubiquitin) by disrupting the production of UBE2N–Ub thioester conjugates. Another inhibitor of UBE2N, BAY 11–7082, modifies the reactive cysteine residues of UBE2N and possibly other E2 enzymes. It effectively inhibits IκB-α phosphorylation in cells [109].

MDM2 is an E3 ligase of the RING type that plays an essential role in modulating the stability of the tumor suppressor protein p53 and preventing cancer. This drug inhibits selectively the growth of cancer cells carrying p53 genes, also known as RITA (NSC652287). Rather than attenuating p53’s transcriptional activity, RITA hinders the interaction between p53 and other regulatory proteins, such as p300, which is responsible for polyubiquitinating p53 with MDM2. RITA induces apoptosis specifically in human tumor cells while having minimal impact on healthy cells. Additionally, it demonstrates a growth-inhibiting effect in a mouse model of tumor xenografts [110, 111].

AT-406, an IAP inhibitor created by Ascenta Therapeutics and the University of Michigan, is now being tested in phase I clinical studies for solid tumors and lymphoma. It is taken orally [112]. MI-63 exhibits significant efficacy in stimulating p53 activity and suppressing the proliferation of cancer cells in cases when p53 is in its natural, unmutated form. Furthermore, it exhibits remarkable selectivity towards cancer cells that lack the p53 gene while demonstrating negligible harm to healthy cells [113].

## Role of PIs toward NF-κB pathway in hematologic malignancies

The hypothetical adverse impacts of PIs could encompass initiation of p53, stimulation of JNK, disruption of NF-κB signaling cascade, genotoxic and oxidative stresses, as well as decline of pro-survival constituents within the BCL-2 lineage [114]. In both in vitro and in vivo settings, PIs exhibit a selective propensity for inducing cytotoxicity specifically in leukemia and lymphoma cells. This phenomenon predominantly arises from the induction of endoplasmic reticulum (ER) stress, whereby the degradation of growth/tumor regulatory proteins and/or misfolded proteins is impeded by the PIs [5, 14, 114, 115]. The inhibition of the proteasome leads to an accumulation of misfolded proteins within the endoplasmic reticulum (ER), consequently activating the unfolded protein response (UPR). This activation is partly facilitated by the ER-associated degradation (ERAD) mechanism, which is responsible for targeting and eliminating misfolded proteins, and the signaling pathway involving protein kinase RNA-like ER kinase (PERK) [114, 116]. Proteasome inhibition also results in intracellular amino acid shortage, which triggers activation of the integrated stress response (ISR) through general control nonderepressible 2 (GCN2) [114]. Both GCN2 and PERK activation contribute to increased expression of activating transcription factor 4 (ATF4) that upregulates the expression of several protein homeostasis genes alongside enhancement of autophagy key genes [114]. Presently, there are only three FDA-approved PIs (BTZ, CFZ, and Ixazomib) used for the treatment of MM and MCL [117, 118].

Blastic plasmacytoid dendritic cell neoplasm (BPDCN), which arises from the myeloid lineage and originates from resting plasmacytoid dendritic cells (pDCs), exhibits an inherent resistance to apoptosis and demonstrates intrinsic resistance to conventional chemotherapeutic agents. Furthermore, BPDCN is characterized by constitutive activation of the NF-κB pathway, which has been established as a potential target for therapy through the use of NF-κB p65 inhibitors, as it has shown sensitivity to their action [119–122].

NF-κB is more active in Ph + ALL and CML blast crisis through Ras signaling, and it plays a key role in its leukemogenesis [123]. Based on reports, BTZ and carfilzomib show efficacious responses in both pediatric and adult ALL, Ph + ALL patients and CML murine model or imatinib-resistant cell lines [90, 124–127].

In acute myeloid leukemia (AML), there are multiple mechanisms involved in the upregulation of NF-κB. One such mechanism involves the interaction between CEBPα and the p50 subset of NF-κB, leading to the induction of upregulation in various components of the Bcl-2 family, FLIP, and tumor necrosis factor α (TNFα) [24, 128]. Furthermore, TNFα binds to the receptor (TNFR) that, via an autocrine mechanism, promotes further IκBα phosphorylation that positively regulates NF-κB [128]. In cases of t(8;21) in AML, there is a specific genetic abnormality involving the translocation of genetic material between chromosomes 8 and 21. This translocation leads to the loss of the c-terminal region of the RUNX1 gene. Consequently, this loss contributes to the activation of IKK and subsequently leads to the activation of NF-κB signaling pathway [129]. In AML cells, FLT3 directly activates IKK and subsequently canonical NF-κB pathway, as well as FL3-ITD that is able to activate the TGF-β-activated kinase 1 (TAK1) that enhances NF-κB activation [130, 131]. Besides, Aurora kinase A (AK-A) is another IKK activator which through TRAF-interacting protein (TIFA), induces IκB degradation [132]. Therapeutic approaches involving BTZ or carfilzomib disrupting this cycle led to autophagy and cell death. After BTZ-based treatment, cytosolic proteins, including FLT3 and TRAF6 within autophagosome vesicles, are delivered to the lysosome for oxidative degradation [128]. It also interferes with C-KIT processing and transforms the t(8;21)-generated fusion proteins into tumor-suppressor fragments in leukemic cells [133].

One of the noticeable effects of NF-κB on AML is histone deacetylase inhibitors (HDACIs) degradation [134, 135]. HDACIs posses tumor suppressor properties, as they facilitate the degradation of specific oncogenic proteins such as FLT3-ITD, AML1-ETO, and PML-RARA. These oncogenic proteins are targeted for destruction through NF-κB-mediated acetylation, which triggers their ubiquitination and subsequent proteasomal degradation within the UPS [134, 135]. Hence, PIs in combination with chemotherapy have been suggested as a possible therapeutic way that may confer more than 80% complete response (CR) in some AML cases; however, many others may not be a responder to this combination [128].

Doxorubicin, an anti-tumor drug, is recognized for its ability to activate the UPS and, consequently, NF-κB. This activation of NF-κB may play a role in the survival of leukemia cells, potentially influencing their ability to evade cell death mechanisms [136, 137]. A study demonstrated that BTZ sensitizes U937 leukemia cells to doxorubicin by suppressing NF-κB and mitochondrial membrane potential loss, which increased apoptosis [136]. Also, the synergy between either arsenic trioxide (ATO) or cytarabine and BTZ in the treatment of acute promyelocytic leukemia is shown to be safe, well-tolerated, less toxic, and more efficacious than single therapy, led to increased overall survival [138, 139]. Additionally, combining a PI with idarubicin was shown to effectively inhibit leukemia initiation by leukemic stem cells (LSCs) without any significant effect on normal CD34+ cells viability or their ability for engraftment in mice model [140, 141]. In general, it suggests that PIs in combination with standard treatments can be regarded for selective targeting of the LSCs and notably an important part of drug resistance in AML relates to LSCs [142]. When LSCs adhere to mesenchymal stromal cells (MSCs) via VLA-4/VCAM-1 axis, NF-κB is activated as an anti-apoptotic factor in both AML LSCs and stromal cells, which promotes the stemness of these cells through LIN28B activation [143, 144]. In primary CD34+/CD38- quiescent AML LSCs, NF-κB is aberrantly activated [11, 145]. It is also betokened that NF-κB activity is increased after chemotherapy while treatment of AML samples with a PI suppressed NF-κB and increased apoptosis selectively in leukemia stem cells, but not in normal hematopoietic stem cells (HSCs) because the NF-κB pathway does not have any significant activity in these cells [11, 140, 146].

In cases of Hodgkin lymphoma (HL), diffuse large B-cell lymphoma (DLBCL), and extranodal natural killer/T-cell lymphoma (ENKTL) that are positive for the Epstein-Barr virus (EBV), the NF-κB and Janus kinase/signal transducer and activator of transcription (JAK/STAT) pathways are continuously active. This activation is aided by the viral protein latent membrane protein 1 (LMP1) [147]. BTZ has demonstrated the ability to inhibit extranodal natural killer/T-cell lymphoma (ENKTL) cells in laboratory studies by inhibiting the NF-κB pathway and inducing cell death through the caspase-mediated pathway [148]. Additionally, BTZ has shown effectiveness against other types of non-Hodgkin lymphomas (NHLs), such as follicular lymphoma (FL) [149–151]. Both canonical and non-canonical NF-κB pathways have shown survival activity in primary and cultured Hodgkin and Reed/Sternberg (HRS) cells of HL [10]. In addition to autocrine and paracrine cytokine loops that can activate NF-κB in HRS cells, mutations in the IκB and A20 genes were also reported to be involved in the aberrant activation of NF-κB in HRS cells [10].

The three main subtypes of diffuse large B-cell lymphoma (DLBCL) are germinal center B-cell like (GCB), activated B-cell like (ABC), and primary mediastinal DLBCL [152]. In both the activated B-cell like (ABC) subtype and primary mediastinal DLBCL subtype of diffuse large B-cell lymphoma (DLBCL), the NF-κB pathway is continuously active. This sustained activation is due to chronic signal transduction from the B-cell receptor (BCR), leading to the upregulation of CARD11, BCL10, and MALT1 [153–156]. The ABC subgroup, which is a more progressive disease and has a poor response, accounts for almost one-third of DLBCL cases [157]. The GCB subtype, which constitutes half of DLBCL cases, is related to C-REL amplification and mutations in BCL-2 and EZH2, known as NF-κB partners [158]. Tonic BCR signaling presents in the GCB DLBCL subtype, which is mechanistically distinct from chronic active BCR signaling since it does not engage BTK, CARD11, or NF-κB [159]. Nonetheless, BTZ induces effective proteasome inhibition and apoptosis by the accumulation of poly-ubiquitinated proteins and ER stress in all of these cells [160].

BTK is also expressed by osteoclasts that contribute to bone destruction in MM. Accordingly, it has been shown if BTK is inhibited using a combination of a Bruton tyrosine kinase inhibitor (CC-292) and a PI (carfilzomib), osteoclasts’ function would be suppressed [161, 162]. The upregulated activity of the proteasome in MM results in excessive degradation of tumor suppressor p53 and IκB. Also, it increases the transcription of NF-κB positive regulators (NIK and NFKB1), as well as TNF receptors (CD40, TACI, LTBR). Intriguingly, as a result, the TNF receptors signaling cascades are activated in the presence of no ligands, which may contribute to the progression of the disease [163, 164].

Inhibition of proteasomes in MM patients has been shown to result in the accumulation of misfolded immunoglobulins (Igs) and provoke the ER stress in MM cells, resulting in cell cycle arrest and apoptosis [115, 165, 166]. However, PI-related preferential cytotoxicity in MM cells correlates with their higher amount of Ig production compared to normal plasma cells [167]. Another beneficial inhibitory NF-κB effect of BTZ may be its ability to downregulation of adhesion molecule ICAM-1 and IL-6 secretion from bone marrow stromal cells (BMSCs) that are known to increase proliferation, survival, and drug resistance in MM cells [168]. All in all, these merits ultimately led to FDA approval of the combined utilization of BTZ with dexamethasone and panobinostat in MM [169].

Since the PI BTZ has demonstrated notable activity in frontline and relapsed/refractory cases of MM and R/R MCL, it also has gained attention as a therapeutic option for patients with Waldenström macroglobulinemia (WM) [94, 170, 171].

PIs have become an important part of both primary and salvage therapy in WM [172]. More than 90% of WM patients carry a mutation in MYD88 (MYD88 L265P) gene [173]. In WM cells, the MYD88 L265P triggers NF-κB through activation of BTK, contributing to proliferation, survival, and Ig production, although it is shown to be inhibited by PIs [172–175]. The induction of ER stress has also been implicated as a mechanism for BTZ activity leading to disruption of the unfolded protein response that prompts apoptosis in both primary and WM cell lines [172]. PIs may also impact the supportive bone marrow microenvironment in WM in combination with other WM-acting drugs [172].

MCL is an NHL subtype with a relatively poor outcome [19]. It has also been betokened that PIs in combination with ara-C, rituximab or cyclophosphamide, HDACIs, and obatoclax, have synergistic effects in MCL cell lines [19]. Of note, PIs have been indicated to imply their anti-tumor effects through different mechanisms, including UPR and ER stress, NF-κB signaling interruption, accumulation of pro-apoptotic proteins, DNA repair dysregulation, and tumor angiogenesis inhibition; however, cases of PI resistance have been reported in MCL [19, 176].

Large granular lymphocyte (LGL) leukemia stems from the clonal proliferation of CD31 positive cytotoxic T cells or CD32 positive natural killer (NK) cells [177]. LGL cells have an upregulated TRAIL expression through which DcR2 mediates constitutive activation of NF-κB [177]. TRAIL binding to death receptors DR4 or DR5 is able to trigger death-induced signaling complex (DISC) formation and ultimately apoptosis via the caspase-8 pathway. While in leukemic cells, it activates the NF-κB pathway through IKK a/b and p65 phosphorylation and TRAF2 pathway [178, 179]. These cells benefit from DcR1, a decoy receptor for TRAIL that competitively inhibits DR4- and/or DR5-associated DISC formation, and DcR2 that prevents TRAIL from DISC formation while mediates it to activate NF-κB [177]. PIs like BTZ or ixazomib effectively interrupt TRAIL-induced activation of NF-κB to downregulate NF-κB–mediated TRAIL gene expression and protein levels in leukemic LGLs to induce apoptosis [177]. In addition, these therapeutic agents decrease anti-apoptotic c-FLIP expression, cell proliferation and induce apoptosis in both LGL leukemia cell lines and primary peripheral blood mononuclear cells through caspase-3 and PARP cleavage [177, 180].

has demonstrated the potential to improve the unfavorable prognosis associated with t(4;14) and del(17p) mutations. These mutations are commonly found in newly diagnosed primary plasma cell leukemia (pPCL) and/or secondary PCL (sPCL). Bortezomib may be effective in these cases by countering the effects of these mutations. Additionally, the abnormal expression of CD27, which significantly increases the activity of ERK1/2 and NF-κB while decreasing JNK signaling, could contribute to the therapeutic efficacy of bortezomib in these contexts [181, 182].

Kaposi’s sarcoma (KS), multicentric Castleman disease (MCD), and primary effusion lymphoma (PEL) have been found to harbor a viral FLICE-inhibitory protein (vFLIP). This vFLIP is a viral counterpart of FLIP, a protein that inhibits apoptosis. In these diseases, vFLIP enhances the activity of the NF-κB pathway, promoting anti-apoptotic signaling pathways. This dysregulated NF-κB activity is believed to play a significant role in the development and progression of these conditions [183]. Furthermore, it has been shown that Kaposi’s sarcoma herpes virus (KSHV) proteins K13 and K15 are involved in the activation of NF-κB in lymphocytes that express the mucosa-associated lymphoid tissue lymphoma translocation 1 (MALT1) protein. This interaction enhances the growth of primary effusion lymphoma (PEL) cells in laboratory studies, suggesting a role for these KSHV proteins in promoting PEL cell proliferation in vitro [184]. Bortezomib (BTZ) has been employed in conjunction with standard chemotherapy agents, such as pegylated liposomal doxorubicin and rituximab, to enhance treatment outcomes in primary effusion lymphoma (PEL) patients. This combination therapy has been reported to extend the duration of remission by up to 2 years. The beneficial effects of BTZ in PEL are attributed to its ability to suppress the NF-κB pathway. Additionally, BTZ exerts proapoptotic effects in PEL cells by inducing cell cycle arrest and downregulating genes associated with DNA replication and Myc signaling [185–188]. Concurrently, the investigations about its safety and effectiveness in refractory/relapsed cases of KS and MCD has been accompanied with very promising results; even when MCD coexisted with MM in one patient [189–192].

Peripheral T-cell lymphomas (PTCL) encompass several subtypes, including PTCL not otherwise specified (NOS), angioimmunoblastic T-cell lymphoma (AITL), ALK-positive anaplastic T-cell lymphoma (ALCL), and ALK-negative ALCL. Unfortunately, these PTCL subtypes are associated with a poor prognosis [193]. However, PIs like BTZ and ixazomib and NF-κB inhibitors have induced noticeable apoptosis in these cells [194–196]. Although BTZ has shown to be safe and increase the OS in these patients and ixazomib was shown to be less effective, it has drawn attention in relapsed/refractory cutaneous T-cell lymphoma (CTCL) and PTCL through inhibition of NF-κB/GATA-3 axis in a clinical trial and was suggested that it might be effective in combination with other therapeutic agents [196–198].

Recurrent mutations in the linker domain of CARD11, as well as alterations in T-cell receptor (TCR) activity, are frequently observed in Mycosis fungoides and Sézary syndrome (MF/SS). These genetic and functional changes play a significant role in the pathogenesis of MF/SS, contributing to the development and progression of these T-cell lymphomas [199]. TCR-dependent and/or phosphorylation of the CARD11 linker domain leads to the CBM complex that constitutively activates the NF-κB pathway in MF/SS malignancies [199], making these cells vulnerable to PIs [199, 200].

BTZ also induces apoptosis in Chronic Lymphocytic Leukemia/Small Lymphocytic Lymphoma (CLL/SLL) cells by enhancing the stability and eliciting the accumulation of the BH3-only protein Noxa [5]. In CLL, which has remained an incurable disease, Btk plays an important role in the survival of these cells [15, 201]. Bone marrow stroma cells, nurse-like cells, and T cells produce chemokines and cytokines that activate Btk, which subsequently activates downstream survival signaling, including extracellular-signal-regulated kinases 1/2 (ERK 1/2), phosphoinositide-3-kinases (PI3K), TNF-a and NF-κB pathway that are constitutively activated in CLL cells and this leads to the transcription and overexpression of key anti-apoptotic proteins [201–203]. Upon the activation of tumor necrosis factor receptor (TNFR), the downstream signaling is triggered by the formation of complex I, which recruits proteins containing the death domain (DD), such as TRADD, FADD, TNF-α receptor associated factor (TRAF) 2/5, cIAP1/2 and RIP1. In complex I, RIP1 is polyubiquitinated by E3 ligase TRAF2/5 and cIAPs and then is able to activate NF-κB essential modulator (NEMO) and IκB kinase (IKK) complex, which promotes the activation of NF-κB pathway, thereby inducing cell survival [203].

In chronic lymphocytic leukemia (CLL), proteasomes play a crucial role in the degradation of regulatory proteins associated with the p53, Bcl-2, and NF-κB families. These regulatory proteins are often abnormally active in CLL. By targeting and breaking down these proteins, proteasomes help regulate their levels and activity, potentially influencing the progression and development of CLL [15]. BTZ remains effective in CLL cells, regardless of their p53 status and in relapsed or treatment-resistant scenarios, as demonstrated in both cell lines and primary samples. The mechanism of action involves inhibiting Bax degradation, which is crucial for CLL cell survival. Consequently, BTZ induces programmed cell death and effectively eliminates CLL cells dependent on blocking Bax degradation [128, 204]. However, this effect was less significant in primary CLL cells when compared to carfilzomib since the cytotoxicity of carfilzomib was mediated by caspase-dependent pathways [15]. However, a clinical trial of BTZ in CLL evidenced several toxic side effects and failed to produce objective responses [205]. These disappointing results may well be related to the fact that BTZ and other PIs (e.g., MG- 132 and epoxomicin) also induce Mcl-1 accumulation, notably in CLL cells: this would decrease the PIs’ apoptotic response and thus therapeutic efficacy [206]. Interestingly, carfilzomib (a second-generation PI) shows activity in CLL cells through an atypical mechanism, which has prompted the initiation of a Phase I clinical study [15]. However, carfilzomib may also promote Mcl-1 upregulation [207].

Carfilzomib, a second-generation PI and a member of the epoxyketone group, exhibits irreversible binding to the b5 subunit of the proteasome. It demonstrates a higher selectivity for the b5 to b2 subunits compared to BTZ. This unique binding property of carfilzomib to both b5 and b2 subunits contributes to its effectiveness in treating plasma cell myeloma that is resistant to BTZ [208, 209]. In contrast to BTZ, carfilzomib demonstrates superior efficacy and is associated with a reduced risk of peripheral neuropathy. It exhibits a higher affinity for the proteasome and has minimal off-target activity beyond the proteasome. Importantly, carfilzomib is capable of inducing apoptosis in MM cells, regardless of their prior exposure to BTZ. This highlights carfilzomib as a promising treatment option for both BTZ-naive and BTZ pre-treated MM patients [116, 209, 210]. However, carfilzomib can only be administrated via intravenous route and has a higher incidence of serious cardiotoxicity, probably due to the reduced number of proteasomes per unit of protein in cardiac muscle and/or the off-target effect of inhibiting autophagy due to activation of protein phosphatase 2A [208–211]. A recent publication confirmed carfilzomib-induced NF-κB inhibition in MM U266 cells; moreover, it demonstrated interesting results involving NF-κB inhibition with curcumin [212].

Ixazomib is a third-generation PI prodrug that belongs to the boronate-based class of drugs. It is specifically approved for the treatment of plasma cell myeloma. Ixazomib can be administered orally, allowing for convenient dosing. Once inside the body, it undergoes hydrolysis to form an active metabolite. This active metabolite binds reversibly to the b5 subunit of the proteasome and, to a lesser extent, to the b1 and b2 subunits. This binding activity contributes to the drug’s mechanism of action in inhibiting the proteasome and exerting its therapeutic effects [98, 116, 209]. Additionally, this unique binding profile allows for better distribution of the drug in the bloodstream and enhanced pharmacodynamic effects in various tissues [116]. However, the time of dissociation from the b5 subunit is shorter for ixazomib when compared with BTZ [98, 116, 209]. Ixazomib effectively inhibits both activation pathways of NF-κB in MM stromal cells, which results in reduced production of MM promoting cytokines and growth factors [213]. Similarly, it was reported that ixazomib decreases the proliferation and survival of myeloma cells while it induces cell cycle arrest, apoptosis, and production of reactive oxygen species (ROS) [214]. It is approved by the FDA for MM treatment and is currently being used in combination with lenalidomide and dexamethasone for relapsed/refractory patients [116]. Because the metabolite is similar to that of BTZ, the incidence of grade 3 or more hematological and gastrointestinal side effects is comparable with BTZ [209]. However, the risk of peripheral neuropathy is lower than that of BTZ [209, 215, 216]. In addition, ixazomib-induced inhibition of NF-κB signaling was also observed in preosteoclasts, which led to reduced osteoclastogenesis and reduced bone destruction [217]. Considering less adverse effects of ixazomib over BTZ, meanwhile being effective in BTZ-resistant cases [169] makes it more ideal in MM cases.

PIs such as BTZ, carfilzomib, ixazomib, oprozomib, delanzomib, and marizomib have the ability to inhibit proteasomes, which results in several beneficial effects in leukemic cells. These inhibitors upregulate JNK (c-Jun N-terminal kinase), FOXO3 (Forkhead box O3), P27 (a cell cycle regulator), and caspase-8 (a key player in apoptosis), while also promoting the accumulation of misfolded proteins. Moreover, they downregulate NF-κB (Nuclear Factor kappa B), a transcription factor involved in cell survival and proliferation. These combined effects ultimately lead to increased apoptosis (programmed cell death) and decreased proliferation of leukemic cells [90, 218]. Caspase-8 activation leads to BID activation, with subsequent activation of BAX and BAD. Moreover, BTZ was able to induce G2/M cell cycle arrest via induction of p27 transcription through promoting transcription activity of CDKN1B as a result of the accumulation of KMT2A fusion proteins upon BTZ treatment [219]. Furthermore, Oprozomib, delanzomib, and marizomib are newer classes of PIs with more favorable side effects profiles [90].

Delanzomib, when compared to BTZ, exhibits a similar potency in inhibiting the proteasome. However, there is a difference in their binding profiles. Delanzomib binds to both the b5 and b1 subunits of the proteasome, while BTZ specifically targets the b5 subunit. Despite this difference in binding specificity, both drugs effectively inhibit proteasome activity, leading to the disruption of protein degradation processes within cells [220]. Delanzomib shows a higher affinity of binding with a 20 times slower rate of dissociation compared with BTZ [221]. Promising outcomes were observed with delanzomib, as it demonstrated the ability to induce apoptosis and inhibit RANKL-induced osteoclastogenesis in both MM (MM) cell lines and cells obtained from patients. These effects were attributed to the drug’s activity in suppressing NF-κB [222]. However, its development was recently discontinued because of its disappointing efficacy results and dose-limiting toxicities [102].

Oprozomib is an epoxyketone similar to that of carfilzomib, and it shows irreversible binding of the b5 subunit. However, the affinity of bindings is higher than that of ixazomib [221]. The most common grade 3 or above adverse events were nausea, vomiting, diarrhea, and thrombocytopenia, and only a rare occurrence of grade 2 or above peripheral neuropathy was reported [223, 224].

Marizomib has the capability to irreversibly bind to the b1, b2, and b5 subunits of the 20S proteasome for an extended period. Safety data from phase I clinical trials demonstrated that hematological toxicities associated with marizomib were less severe compared to those observed with BTZ and carfilzomib [209, 225]. Common adverse events associated with marizomib treatment include nausea, diarrhea, and fatigue. Some patients experienced central nervous system toxicities, such as reversible hallucinations and cognitive decline. However, cardiac events were less frequent compared to BTZ and carfilzomib, and no patients experienced grade 3 or higher peripheral neuropathy during treatment [226, 227].

## Limitations of PIs

Resistance to the PI BTZ has been observed, and in human AML cells has been reported to be unrelated to the presence of multi-drug resistance (MDR) genes such as P-glycoprotein 1/ATP-binding cassette sub-family B member 1 (P-gp1/ABCB1), multi-drug resistance protein 1 (MRP1/ABCC1), Breast Cancer Resistance Protein (BCRP/ABCG2), and lung resistance-related protein (LRP). However, despite the absence of these MDR genes, leukemic cells can still develop resistance to PIs. This suggests that various reseans are at play in the development of resistance to PIs in leukemic cells [228].

### Interruption of chemotherapy

The combined treatment of acute promyelocytic leukemia (APL) cells with arsenic trioxide (ATO) and all-trans retinoic acid (ATRA) has been shown to rely on the degradation of Nucleophosmin 1 (NPM1) by proteasomes. NPM1 mutations are the most common mutations found in AML. Moreover, the degradation of NPM1 is essential for inducing apoptosis in APL cells during this combined treatment [128, 229, 230]. Therefore, the use of PIs in such cases may interfere with the treatment, as it could prevent the necessary degradation of NPM1 and potentially hinder the induction of apoptosis [128].

### Mutations in proteasomes

PIs primarily act on the b5 subunit of the proteasome, which can undergo genetic changes such as point mutations or gene amplifications, resulting in its increased expression in certain situations [231–233]. Upregulation of the b5 augments chymotrypsin activity and thus NF-κB, which is a possible mechanism of resistance in T-ALL cell lines [234]. On the other hand, T-ALL cells that exhibit resistance to BTZ have been observed to have reduced levels of interferon-gamma (IFN-γ), which is an inducer of immunoproteasomes. However, treatment with IFN-γ has been shown to restore sensitivity to BTZ and carfilzomib in these resistant cells [90, 235, 236]. The upregulation of PSMA1, a crucial subunit of the proteasome, has been found to contribute to resistance to BTZ. Conversely, the inhibition of TRAF6 has been shown to restore sensitivity in resistant cell lines [237]. It is noteworthy that these mutations or alterations are not commonly observed in MM patients [114].

### Alternative protein degrading systems

Cellular proteolytic systems can be categorized into four main groups: membrane proteases, mitochondrial proteases, proteasomal proteases, and lysosomal proteases [2]. One protein with a short lifespan, IκBα, can undergo degradation not only through the proteasome system but also through autophagy in MM cells. This suggests that the degradation of IκBα can occur through multiple pathways, highlighting the complexity of protein turnover in MM cells [238–240].

MM cells can escape proteasome inhibition by activating the autophagy pathway, which highlights the importance of targeting autophagy in this disease [114, 241]. However, results of a clinical trial using a combination of hydroxychloroquine and BTZ, as well as proteasome and autophagy inhibitors in relapsed/refractory MM, were not promising [242, 243].

### Intrinsic NF-κB pathway up regulators

Malignant cells and LSCs can develop resistance to BTZ by upregulating NF-κB, increasing the expression of MCL-1, or altering metabolic pathways to enhance mitochondrial activity. However, the addition of a pan Bcl-2 inhibitor, such as obatoclax, can overcome this resistance and restore sensitivity to BTZ in these cells [128, 244, 245]. The mechanism involves the participation of Zinc Fingers and Homeoboxes 2 (ZHX2), Nuclear factor erythroid 2-related factor 2 (Nrf2), and heme oxygenase-1 (HO-1) in mediating the resistance process [246–248]. ZHX2 degradation is shown to be proteasome-mediated and is upregulated after BTZ treatment [246]. It directly enhances the nuclear translocation of NF-κB in MM cell lines, including RPMI 8226 and MM.1S [246]. Hence, ZHX2 counteracts the anti-tumor activity of PIs in resistant cases, and its higher expression is accompanied by less promising clinical outcomes in MM patients [246]. Furthermore, overexpression of HO-1 and Nrf2 transcription factors mediates BTZ resistance in leukemic cells in response to BTZ-ROS through decreasing Bach1 and increasing HO-1 which contributes to the protection of leukemic cells against ROS formation, NF-κB inhibition, and chemotherapy against apoptosis [247, 248].

### Flavonoids

Flavonoids present in human serum have been found to neutralize the activity of BTZ in CLL patients and CLL cell lines cultured in media containing human serum. This phenomenon helps explain why BTZ does not exhibit significant activity in CLL patients, despite its notable efficacy in in vitro studies [15, 205, 249]. These findings suggest that BTZ may not effectively inhibit NF-κB in CLL cells due to the neutralizing effects of flavonoids present in human serum.

### Dose-limiting toxicities

In certain cases, the acquired or primary resistance to PIs can lead to a required minimum inhibitory concentration that exceeds the therapeutic benefits. Consequently, the side effects associated with PIs may outweigh their advantages. This can result in suboptimal concentrations of PIs, limiting their ability to effectively suppress NF-κB signaling. For example, the plasmacytic differentiation of MCL cells can significantly increase resistance to BTZ. Effective doses of PIs, particularly when combined with rituximab, may lead to peripheral neuropathy, which is a significant adverse effect [250, 251].

### Interaction with tumor microenvironment

According to Kuroda et al., BTZ was found to induce apoptosis in MCL cell lines. However, in murine models or when MCL cells were co-cultured with stromal cells, a process involving p62-mediated autophagic degradation of the pro-apoptotic protein NOXA from the BCL-2 family was activated. This activation was attributed to the secretion of IL-6 by stromal cells, which conferred resistance to PIs [252]. The IL-6 prevents ubiquitinated NOXA from inducing mitochondrial damage and subsequently apoptosis; instead, it activates p62 and LC3-II to destroy ubiquitinated NOXA in lysosomes, which increases STAT3 activation and/or NF-κB nuclear translocation [252, 253]. MM cells have been shown to follow a similar pattern [254], and thus administration of anti-IL-6 antibody may be a solution for PI-resistance in MM or MCL and to down-regulate NF-κB in these cells.

### PIs may independently activate NF-κB in cancer cells

Research findings have indicated that in certain cases, BTZ can directly activate the NF-κB pathway along with its downstream genes. This activation occurs through the downregulation of IκB expression [168, 255]. Like BTZ, carfilzomib and oprozomib have the ability to upregulate the anti-apoptotic protein MCL1. However, the impact of this upregulation can be mitigated by the use of an MCL1 inhibitor such as obatoclax [256]. Additionally, BTZ treatment has been shown to induce phosphorylation and ubiquitination of IκBα, leading to its degradation through a non-proteasomal pathway. This degradation of IκBα contributes to NF-κB activation via the autophagy pathway [21, 160, 239]. Notably, it has been observed that inhibition of autophagy alone can actually enhance the phosphorylation of IκBα [160], underscoring the existence of cross-talk between these two pathways and their involvement in cytoprotective mechanisms [160].

As mentioned before, PIs cause ER stress through ROS generation and accumulation of misfolded proteins. This leads to the release of C/EBP Homologous Protein (CHOP) protein from the ER, resulting in increased cellular calcium levels. Elevated calcium levels activate calpain, which promotes autophagy and activates the caspase-8/caspase-3/PARP-1 axis [257]. Nevertheless, autophagy is shown to be able to inhibit caspases and increase cell survival [258]. In the context of BTZ-induced autophagy, p62 interacts with microtubule-associated protein light chain 3-II (LC3-II) to facilitate the targeting of ubiquitinated proteins, including IκBα, to the autophagosome. This process leads to the activation of canonical NF-κB signaling and the expression of downstream genes [160, 259]. However, this does not prevent the accumulation of p53 and pro-apoptotic Bax in BTZ-treated cells [239].

In a study involving DLBCL cell lines (DoHH2, Su-DHL4, and Su-DHL10) and primary cells from FL patients, it was observed that BTZ-induced autophagy could contribute to drug resistance. However, when autophagy inhibitors such as chloroquine were combined with BTZ, a synergistic effect was observed, leading to an increased activation of the mitochondrial apoptosis pathway in drug-resistant cells [160].

Markovina and colleagues showed that BTZ may fail NF-κB suppression in MM cell lines [260]. This was later confirmed by Hideshima and colleagues later who reported that BTZ promotes non-proteasomal degradation of IκB through activation of IKK and RIP2, leading to improved canonical NF-κB activity in both MM cell lines and primary cells [21]. Moreover, Li and colleagues suggested that BTZ can activate NF-κB activity by calpain-mediated IκB degradation and increased p65 nuclear translocation [21, 238]. In a similar way, carfilzomib is also reported to activate NF-κB through an atypical or calpain-mediated NF-κB pathway in CLL cells which is IKK-independent and directly promotes p50/p65 nuclear translocation [15]. In this pathway, tyrosine kinase, as well as casein kinase II (CK2), play a central role in activating calpain that can phosphorylate IκB [261, 262]. All FDA-approved PIs, including BTZ, carfilzomib, and Ixazomib, have been shown to increase the levels of ROS and induce oxidative stress. This elevation of ROS and oxidative tension is considered a crucial factor in the cell death mechanism mediated by PIs [160, 214, 263]. Notably, ROS is further increased following PI treatment in PI-resistant malignant cells without any significant effect on oxidative damage in these cells [264]. ROS can activate various cellular signaling pathways, including the activation of protein kinases such as CK2 and tyrosine kinases. Additionally, ROS can contribute to the activation of IKK by recruiting NF-κB-inducing kinase (NIK) and facilitating the phosphorylation of JNK, P38, MAPK, and ERK. Moreover, ROS can induce genotoxic damage, leading to nuclear translocation, ubiquitination, and sumoylation of NEMO (NF-κB essential modulator), which further activates IKK. This cascade of events ultimately promotes NF-κB signaling and its downstream effects [265–271]. In a study conducted by Gupta et al., it was demonstrated that carfilzomib treatment in CLL primary cells had the unexpected effect of inducing the NF-κB pathway instead of inhibiting it. The cells exposed to carfilzomib exhibited activation of the non-canonical NF-κB pathway and the expression of specific target genes such as CXCL13, c-FLIP, and IL-6. However, there was no observed induction of classic NF-κB target genes, including Bcl2A1, XIAP, Mcl-1, and p53 [15]. Although the NF-κB pathway was induced by carfilzomib, its cytotoxic effects on CLL patient cells were intact, and no NF-κB-induced resistance to this agent was seen.

All in all, studies have revealed that PIs can have complex effects on the NF-κB pathway, involving both activation and inhibition. Understanding these mechanisms can help in optimizing the therapeutic potential of PIs. Further research is needed to explore the interplay between PIs, NF-κB, autophagy, and ROS, with a focus on identifying strategies to overcome drug resistance and improve treatment outcomes. Future perspectives include the development of combination therapies targeting specific components of these pathways to enhance the efficacy of PIs in cancer treatment.

## Conclusion and future prospective

In conclusion, the NF-κB pathway plays a critical role in the pathogenesis of leukemia and lymphoma, making it an attractive target for cancer therapy. PIs have emerged as promising agents for inhibiting the NF-κB pathway and inducing apoptosis in cancer cells. However, the efficacy of PIs is still limited due to various factors, such as off-target effects and drug resistance. Therefore, it is crucial to continue investigating the underlying mechanisms of PIs and their interactions with the NF-κB pathway.

Future prospective studies could focus on developing more potent and specific PIs that can selectively inhibit the NF-κB pathway in cancer cells. Furthermore, combining PIs with other chemotherapeutic agents or immunotherapies could enhance their therapeutic effects and overcome drug resistance. Finally, identifying predictive biomarkers for PI responsiveness could help to identify patients who are most likely to benefit from this treatment approach. Overall, further research in this field holds great promise for advancing our understanding of PIs and their potential clinical applications in the treatment of leukemia and lymphoma.

## Data Availability

Not applicable.
